# Clinical Characteristics and Prognosis of 218 Patients With COVID-19: A Retrospective Study Based on Clinical Classification

**DOI:** 10.3389/fmed.2020.00485

**Published:** 2020-08-11

**Authors:** Xiquan Yan, Xiaotong Han, Danhong Peng, Yong Fan, Zhixiong Fang, Da Long, Yu Xie, Shuibo Zhu, Fang Chen, Wei Lin, Yimin Zhu

**Affiliations:** ^1^Hunan Provincial Institute of Emergency Medicine, Hunan Provincial People's Hospital/The First Affiliated Hospital, Hunan Normal University, Changsha, China; ^2^School of Life Sciences, Hunan Normal University, Changsha, China; ^3^Xiangtan Central Hospital, Xiangtan, China; ^4^Department of Infectious Diseases, Shaoyang Central Hospital, Shaoyang, China; ^5^Department of Infectious Diseases, Loudi Central Hospital, Loudi, China; ^6^Public Health Centre, Xiangtan Central Hospital, Xiangtan, China; ^7^Department of Science and Education, Shaoyang Central Hospital, Shaoyang, China; ^8^Department of Critical Care Medicine, Loudi Central Hospital, Loudi, China; ^9^Department of Hepatobiliary Surgery, Loudi Central Hospital, Loudi, China

**Keywords:** COVID-19, clinical classification, clinical characteristics, treatment, prognosis

## Abstract

**Background:** Coronavirus disease 2019 (COVID-19) is an emerging infectious disease that has spread worldwide.

**Methods:** This was a retrospective case series involving 218 patients admitted to three tertiary hospitals in the Loudi, Shaoyang, and Xiangtan areas of China from January 21 to June 27, 2020, who were confirmed by RT-PCR to have SARS-CoV-2. The patients' clinical characteristics, laboratory results, treatments, and prognoses based on clinical classification were recorded. Poor outcome was defined as admission to an ICU, the use of mechanical ventilation, or death.

**Results:** The patients were classified into four clinical groups based on disease severity, namely mild (10/218, 5%), moderate (146/218, 67%), severe (24/218, 11%), or critical (14/218, 6%); 24 (11%) asymptomatic cases were also included in the study. The most common symptoms were self-reported cough (162/218, 74%), fever (145/218, 67%), sputum production (99/218, 45%), and fatigue (77/218, 35%). Among the 218 patients, 192 (88%) received lopinavir/ritonavir and interferon-alpha inhalation, and 196 (90%) patients received traditional Chinese medicine. Among the severe and critical patients, 25 (11%) were admitted to an ICU with or without mechanical ventilation, and one patient died. The presence of diabetes [relative risk (RR), 3.0; 95% CI, 1.3–6.8; *p* = 0.007) or other comorbidities (RR, 5.9; 95% CI, 1.9–17.8; *p* = 0.002) was independently associated with poor outcome. To date, 20 (9%) patients have retested positive for SARS-CoV-2 RNA after recovering and being discharged.

**Conclusion:** The majority of patients in this case series were clinically classified as having moderate COVID-19. Older patients tended to present with greater levels of clinical severity. The prognosis for patients who were elderly or had diabetes or other chronic comorbidities was relatively poor.

## Introduction

Coronavirus disease 2019 (COVID-19) is caused by the severe acute respiratory syndrome coronavirus-2 (SARS-CoV-2) and has rapidly spread across the world since first emerging in December 2019 (1). By April 17, 2020, COVID-19 had been discovered in 212 countries or territories, affecting 2,074,529 individuals and causing 139,378 deaths (2). The pandemic continues to escalate rapidly (3, 4). Typical symptoms are fever, cough, fatigue, and sputum production (5–7). However, a few patients with SARS-CoV-2 develop severe pneumonia, pulmonary edema, acute respiratory distress syndrome (ARDS), multiple organ failure, or even death (8–10).

In this retrospective case series, 218 patients testing positive for SARS-CoV-2 were clinically classified (mild, moderate, severe, or critical) according to the guidelines of the *Diagnosis and Treatment Protocol for COVID-19 (trial version 7)* issued by the National Health Commission of the People's Republic of China (11). Asymptomatic patients, who acquire and can transmit the coronavirus that causes COVID-19 (12, 13), were also included in this study.

These clinical classifications of COVID-19 are characterized by different clinical features and provide an objective basis for treatment and prognosis. To date, there have been no studies reporting COVID-19 treatment and outcomes based on clinical classification. Here, we comprehensively explored the clinical features, treatment, and prognosis of 218 confirmed SARS-CoV-2-infected patients in three top-tier hospitals in the Hunan province of China.

## Materials and Methods

### Study Design and Participants

This multicenter, retrospective, and observational study was conducted on COVID-19 patients who were diagnosed in the Hunan province of China. Clinicians collected the patients who met the study inclusion criteria across three tertiary hospitals in the cities of Shaoyang, Loudi, and Xiangtan. The authors of this paper include the physicians who either supervised patient care or directly provided patient care for all of the patients included in the study to ensure complete follow-through for all cases.

We retrospectively analyzed COVID-19 patients who had been diagnosed during the period of January 21 to June 27, 2020, according to the WHO interim guidance. Real-time, reverse-transcription polymerase chain reaction (RT-PCR) tests for SARS-CoV-2 nucleic acids were performed on nasopharyngeal swabs from suspected patients to confirm the diagnosis. A confirmed case of COVID-19 was defined as having a positive result from the RT-PCR assay of a nasopharyngeal swab. Only laboratory-confirmed cases were included in the analysis. Suspected patients showing negative results after multiple tests during hospitalization were excluded. Where the typical symptoms, signs, and imaging manifestations were present, combined with a PaO_2_/FiO_2_ ratio <300 mmHg [based on the Berlin definition (14)], the patients were diagnosed as having ARDS. This study was approved by the ethics committee of each participating hospital. Written informed consent was obtained from all patients.

### Clinical Classification

In this retrospective study, the whole disease course was examined for each patient. The clinical classification of the patients was based on the clinical conditions present during the most severe stage of COVID-19 based on the guidelines outlined in the *Diagnosis and treatment protocol for COVID-19* (trial version 7) released by the National Health Commission of the People's Republic of China on March 3, 2020 (http://www.nhc.gov.cn/xcs/zhengcwj/202003/46c9294a7dfe4cef80dc7f5912eb1989.shtml) (11). According to their clinical symptoms, signs, and chest imaging manifestations, the patients were classified as being mild, moderate, severe, or critical COVID-19 cases (see Supplementary Material for further details).

### Data Collection

Data on the clinical characteristics, treatment, and prognosis of the 218 confirmed COVID-19 patients were collected at Shaoyang Central Hospital, Loudi Central Hospital, and Xiangtan Central Hospital in the Hunan province. The information of interest included age, sex, exposure history, smoking history, chronic diseases (including diabetes), symptoms from onset to hospital admission, laboratory tests on admission, coexisting infections, treatment, and living status. The data regarding the PaO_2_/FiO_2_ ratios were analyzed when the patients were monitored in the ICU.

### Treatment

The patient treatment venue was determined based on the severity of each patient's disease according to the *Diagnosis and treatment protocol for COVID-19* (11). Suspected and confirmed cases were isolated and treated at designated hospitals with effective isolation, protection, and prevention conditions. Suspected cases were treated in isolation or together in a single room. Confirmed cases were treated in isolation or together in a single room. In the absence of pathogen-specific interventions, patient management largely depended on supportive treatment.

Most patients were provided with effective oxygen therapy, including a nasal catheter, mask oxygenation, and nasal high-flow oxygen therapy. Lopinavir/ritonavir, interferon-alpha inhalation, and arbidol were used as antiviral therapies. Moxifloxacin and other antibiotics were used to fight against bacterial infections where present. Glucocorticoids were used for short periods when patients showed rapidly progressive deterioration.

Patients who met the following criteria were admitted to the ICU for comprehensive treatment and care at an early stage: (1) severe cases with respiratory distress (≥30 breaths/min) and chest imaging showing >50% of lung area with obvious lesion progression within 24–48 h; and (2) all critical cases.

In addition, patients were treated with traditional Chinese medicine (Qingfei Paidu decoction, Lianhuaqingwen capsules, Huoxiangzhengqi liquid, and/or Xuebijing injection) according to the national guidelines. The full treatment protocol used for the COVID-19 patients is described in detail in the Supplementary Materials.

### Discharge

When a patient's body temperature had returned to normal for more than 3 days, respiratory symptoms were significantly improved, pulmonary imaging showed obvious absorption of inflammation, and two consecutive SARS-CoV-2 nucleic acid tests were negative using respiratory tract samples (sampling interval of at least 24 h), he or she was discharged from the hospital. After discharge, the patients were required to quarantine and monitor their health for 14 days and requested to come back to the hospital for follow-up exams every 2–4 weeks.

### Prognosis

All patients were traced from hospital admission to presenting prognosis. The primary outcome was “cured and discharged,” and a poor outcome was defined as admission to an ICU, the use of mechanical ventilation, or death. This analysis method was referenced from other retrospective studies on viral pneumonia, such as SARS (15, 16). Time to discharge, time to death, and time to a poor outcome were analyzed using survival analysis (details in *Statistical Analysis*) tracing all patients from hospital admission to presenting prognosis.

### Statistical Analysis

Data are presented as the mean ± standard deviation (SD) or median ± interquartile range (IQR) for continuous variables and as a number (%) for categorical variables. Differences in measurement data among the asymptomatic, mild, moderate, severe, and critical cases were compared with analysis of variance using the least significant difference *post-hoc* test. The Chi-square test and Fisher's exact test were used for categorical variables. Kaplan–Meier plots were used to analyze the survival data. Differences among groups of time-to-event data were determined using the Cox proportional hazards model, with graphical and statistical checks for the proportionality of hazards. Given that there were only 25 patients with poor outcomes in our study, we considered only three binary variables in the multiple regression model as *a priori* hypotheses: age of 60 years or older, diabetes, and other comorbidities. We used SPSS (version 26.0) for all analyses. For all analyses, *p* < 0.05 was considered statistically significant.

## Results

### Demographics

**Figure 1:**
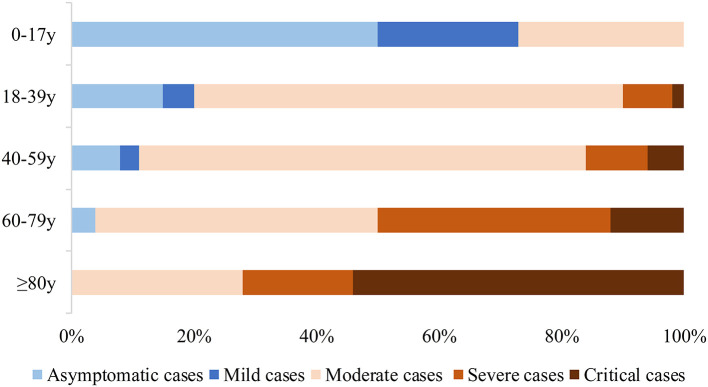
Clinical classification (including asymptomatic cases) and age distribution of patients with COVID-19.

A total of 218 patients were confirmed during the study period. The patients' demographic details and comorbidities are listed in Table 1. Age was correlated with the clinical classification of COVID-19 severity (Figure 1). The median age of the patients was 43 years (IQR 32–52), with 14 (6%) patients <18 years of age and 6 (3%) ≥80 years old; 122 (56%) were male. A total of 100 patients (46%) had known exposure to COVID-19, and 111 patients (51%) had recently traveled to Wuhan, China. There were four (2%) patients who had neither traveled recently to Wuhan nor had known exposure to confirmed COVID-19 patients who were nevertheless diagnosed with COVID-19, and the route of transmission in these cases might have been the use of public transportation. As for their personal medical history, 23 (11%) patients had a history of smoking, 38 (17%) had cardiovascular disease, 27 (12%) had diabetes, 14 (6%) had chronic pulmonary disease, 13 (6%) had liver disease, 10 (5%) had nutritional deficiency diseases, six (3%) had cerebrovascular disease, four (2%) had chronic renal diseases, two (1%) had cancer, and two (1%) had autoimmune diseases.

**Table 1:** Demographics and baseline characteristics of patients with COVID-19.

	**All patient (*n =* 218)**	**Asymptomatic cases (*n =* 24)**	**Mild cases (*n =* 10)**	**Moderate cases (*n =* 146)**	**Severe cases (*n =* 24)**	**Critical cases (*n =* 14)**	***P*-value**
Age, years	42.9 (32.0–52.3)	32.0 (16.3–44.8)^c, d, e^	23.6 (11.8–34.3)^c, d, e^	42.3 (32.0–50.0)^a, b, d, e^	55.9 (46.3–67.0)^a, b, c^	59.5 (42.3–76.5)^a, b, c^	0.000*
**Age range, years**							
0–17	14 (6%)	6 (25%)	4 (40%)	4 (3%)	0	0	0.000**
18–39	82 (38%)	11 (46%)	5 (50%)	60 (41%)	4 (17%)	2 (14%)	
40–59	86 (39%)	6 (25%)	1 (10%)	65 (45%)	9 (37%)	5 (36%)	
60–79	30 (14%)	1 (4%)	0	15 (10%)	10 (42%)	4 (29%)	
≥80	6 (3%)	0	0	2 (1%)	1 (4%)	3 (21%)	
**Sex**							
Male	122 (56%)	16 (67%)	6 (60%)	77 (53%)	14 (58%)	9 (64%)	0.691
Female	96 (44%)	8 (33%)	4 (40%)	69 (47%)	10 (42%)	5 (36%)	
**Exposure**							
Exposure to Wuhan	111 (51%)	12 (50%)	3 (30%)	76 (52%)	13 (54%)	7 (50%)	0.768
Exposure to patients^†^	100 (46%)	18 (75%)	7 (70%)	59 (40%)	8 (33%)	8 (57%)	0.006**
Use of public transportation^‡^	4 (2 %)	0	0 (%)	3 (2%)	0	1 (7%)	0.535
Current smoking	23 (11%)	2 (8%)	2 (20%)	14 (10%)	3 (13%)	2 (14%)	0.902
**Chronic medical illness**							
Cardiovascular disease	38 (17%)	3 (13%)	0	17 (12%)	13 (54%)	5 (36%)	0.000**
Diabetes	27 (12%)	3 (13%)	0	12 (8%)	10 (42%)	2 (14%)	0.001**
Chronic pulmonary disease	14 (6%)	0	0	5 (3%)	4 (17%)	5 (36%)	0.000**
Liver disease	13 (6%)	1 (4%)	0	10 (7%)	2 (8%)	0	0.909
Malnutrition^§^	10 (5%)	0	1 (10%)	6 (4%)	1 (4%)	2 (14%)	0.193
Cerebrovascular disease	6 (3%)	0	0	2 (1%)	1 (4%)	3 (21%)	0.014**
Chronic renal diseases	4 (2%)	0	0	1 (1%)	1 (4%)	2 (14%)	0.031**
Cancer	2 (1%)	0	0	2 (1%)	0	0	1.000
Autoimmune disease	2 (1%)	0	0	1 (1%)	0	1 (7%)	0.256

*Data are median (IQR), n (%), or mean (SD), unless otherwise specified*.

* *ANOVA was used for group comparisons with LSD for post-hoc tests*.

*^a^vs. Asymptomatic cases (p < 0.05), ^b^vs. Mild cases (p < 0.05), ^c^vs. Moderate cases (p < 0.05), ^d^vs. Severe cases (p < 0.05), ^e^vs. Critical cases (p < 0.05)*.

** *Statistical analysis was performed with the chi-square test or Fisher's exact test*.

† *Patients who have confirmed SARS-CoV-2 infection or are highly suspected of being infected*.

‡ *Without exposure to Wuhan and diagnosed patients*.

§ *In this cohort, 3 patients suffer from undernutrition and 7 are overweight*.

### Disease Course

**Figure 2:**
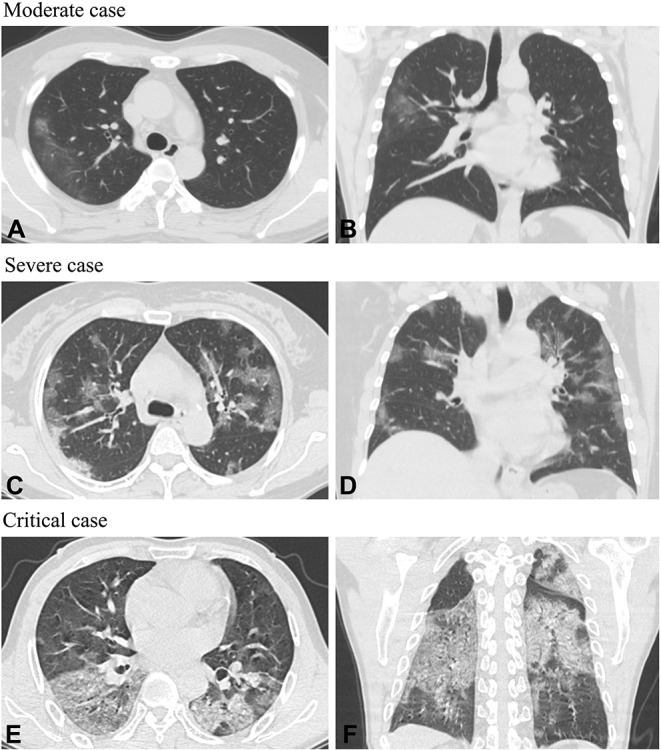
Axial planes and coronal chest CT scans in patients with COVID-19. Moderate case: **(A,B)** Chest CT images of a 41-year-old man showed a ground-glass lesion in the right lobe on the 3rd day following a fever. Severe case: **(C,D)** Chest CT images of a 55-year-old woman showed bilateral multifocal ground-glass opacities on the 8th day after having chills, cough, and expectoration. Critical case: **(E,F)** Chest CT images of a 61-year-old man showed diffuse patchy shadowing and mixed consolidation on the 13th day after having cough, expectoration, and fever.

The patients' COVID-19 onset symptoms are shown in Table 2. Common clinical features included cough (162/218, 74%), fever (145/218, 67%), sputum production (99/218, 45%), and fatigue (77/218, 35%). Only 3% (6/218) of patients had nasal congestion and rhinorrhea. On admission, 39% (86/218) of patients had a recorded temperature of ≥38.1°C. No lung lesions were identified in the computed tomography (CT) scans of asymptomatic and mild cases. In moderate cases, the main imaging changes were ground-glass opacities and local patchy shadowing. In severe cases, the principal abnormality visible on CT scans was diffuse patchy shadowing. In critical cases, pulmonary consolidation and diffuse patchy shadowing were more common (Table 3). Several of the characteristic chest CT features of COVID-19 observed in the moderate, severe, and critical cases are shown in Figure 2. Although there was a notable degree of variability in the pattern of the infiltrates (ground-glass, local, diffuse, pulmonary consolidation), most patients had ground-glass opacities.

**Table 2:** Symptoms (at the time of admission), comorbidities, treatments, and prognosis of patients with COVID-19.

	**All patients (*n =* 218)**	**Asymptomatic cases (*n =* 24)**	**Mild cases (*n =* 10)**	**Moderate cases (*n =* 146)**	**Severe cases (*n =* 24)**	**Critical cases (*n =* 14)**	***P*-value**
**Symptoms**^†^							
Fever	145 (67%)	0	2 (20%)	108 (74%)	23 (96%)	12 (86%)	0.000**
<37.3°C	73 (33%)	24 (100%)	8 (80%)	38 (26%)	1 (4%)	2 (14%)	
37.3–38.0°C	59 (27%)	0	2 (20%)	45 (31%)	8 (33%)	4 (29%)	
38.1–39°C	70 (32%)	0	0	55 (38%)	11 (46%)	4 (29%)	
>39°C	16 (7%)	0	0	8 (5%)	4 (17%)	4 (29%)	
Cough	162 (74%)	0	10 (100%)	117 (80%)	21 (88%)	14 (100%)	0.000**
Sputum production	99 (45%)	0	4 (40%)	68 (47%)	15 (63%)	12 (86%)	0.018**
Fatigue	77 (35%)	0	1 (10%)	55 (38%)	12 (50%)	9 (64%)	0.006**
Shortness of breath	42 (19%)	0	0	16 (11%)	16 (67%)	10 (71%)	0.000**
Myalgia	41 (19%)	0	0	32 (22%)	6 (25%)	3 (21%)	0.407
Chills	39 (18%)	0	0	23 (16%)	9 (38%)	7 (50%)	0.001**
Headache	28 (13%)	0	1 (10%)	18 (12%)	4 (17%)	5 (36%)	0.125
Sore throat	25 (11%)	0	1 (10%)	20 (14%)	2 (8%)	2 (14%)	0.942
Diarrhea	16 (7%)	0	1 (10%)	11 (8%)	3 (13%)	1 (7%)	0.722
Nasal congestion and rhinorrhea	6 (3%)	0	0	6 (4%)	0	0	1.000
**Complications**							
ARDS	14 (6%)	0	0	0	0	14 (100%)	0.000**
Liver dysfunction	40 (18%)	0	1 (10%)	23 (16%)	9 (38%)	7 (50%)	0.000**
Acute kidney injury	10 (5%)	0	0	4 (3%)	1 (4%)	5 (36%)	0.001**
Acquired pneumonia	20 (9%)	0	0	3 (2%)	4 (17%)	13 (93%)	0.000**
Septic shock	4 (2%)	0	0	0	0	4 (29%)	0.000**
**Treatment**							
Oxygen treatment^‡^	156 (72%)	0	3 (30%)	115 (79%)	24 (100%)	14 (100%)	0.000**
Mechanical ventilation	16 (7%)	0	0	0	2 (8%)	14 (100%)	0.000**
Non-invasive	9 (4%)	0	0	0	2 (8%)	7 (50%)	
Invasive	7 (3%)	0	0	0	0	7 (50%)	
Prone position ventilation	14 (6%)	0	0	0	0	14 (100%)	0.000**
Renal replacement therapy	5 (2%)	0	0		11 (46%)	3 (21%)	0.000**
Convalescent plasma	4 (2%)	0	0	0	0	4 (17%)	0.000**
Stem cell treatment	3 (1%)	0	0	0	0	3 (21%)	0.000**
Lopinavir/ritonavir	192 (88%)	19 (79%)	7 (70%)	133 (91%)	20 (83%)	13 (93%)	0.172
Interferon alpha inhalation	192 (88%)	18 (75%)	7 (70%)	131 (90%)	23 (96%)	13 (93%)	0.059
Arbidol	126 (58%)	9 (38%)	3 (30%)	83 (57%)	18 (75%)	13 (93%)	0.001**
Antibiotics	115 (53%)	6 (25%)	1 (10%)	71 (49%)	23 (96%)	14 (100%)	0.000**
Chinese medicine^§^	196 (90%)	21 (88%)	9 (90%)	133 (91%)	20 (83%)	13 (93%)	0.714
Qingfei Paidu decoction	114 (52%)	17 (71%)	6 (60%)	75 (51%)	9 (38%)	7 (50%)	0.220
Lianhuaqingwen capsule	66 (30%)	3 (13%)	2 (20%)	47 (32%)	10 (42%)	4 (29%)	0.203
Huoxiangzhengqi liquid	6 (3%)	0	0	6 (4%)	0	0	0.822
Xuebijing injection	26 (12%)	0	0	11 (8%)	7 (29%)	8 (33%)	0.000**
Corticosteroid	47 (22%)	0	0	17 (12%)	18 (75%)	12 (86%)	0.000**
Gamma globulin	33 (15%)	0	0	13 (9%)	11 (46%)	9 (64%)	0.000**
**Prognosis**							
Discharge from hospital	217 (99.5%)	24 (100%)	10 (100%)	146 (100%)	24 (100%)	13 (93%)	0.000**
Death	1 (0.5%)	0	0	0	0	1 (7%)	
**The hospitalization days of discharged patients**	12.2 ± 6.2	7.1 ± 2.8^c, d, e^	8.6 ± 5.0^d, e^	12.1 ± 5.8^a, d, e^	16.1 ± 5.5^a, b, c, e^	20.5 ± 6.0^a, b, c, d^	0.000*

*Data are mean (SD) or n (%)*.

* *ANOVA was used for group comparisons with LSD for post-hoc tests*.

*^a^vs. Asymptomatic cases (p < 0.05), ^b^vs. Mild cases (p < 0.05), ^c^vs. Moderate cases (p < 0.05), ^d^vs. Severe cases (p < 0.05), ^e^vs. Critical cases (p < 0.05)*.

** *Statistical analysis was performed with the chi-square test or Fisher's exact test*.

† *In part of this series, asymptomatic cases were not included in the statistics*.

‡ *Oxygen therapy includes nasal catheter, mask oxygenation and nasal high-flow oxygen therapy*.

§ *A small number of patients were given two or more kind of Chinese medicine*.

**Table 3:** Chest CT/X-ray features of patients with COVID-19 at the most severe stage.

**Distribution of pulmonary lesions**	**All patients (*n =* 218)**	**Asymptomatic cases (*n =* 24)**	**Mild cases (*n =* 10)**	**Moderate cases (*n =* 146)**	**Severe cases (*n =* 24)**	**Critical cases (*n =* 14)**
No lesion	37 (17%)	24 (100%)	10 (100%)	3 (2%)	0	0
Ground-glass opacities	65 (30%)	0	0	65 (45%)	0	0
Local patchy shadowing	77 (35%)	0	0	76 (52%)	1 (4%)	0
Diffuse patchy shadowing	30 (14%)	0	0	2 (1%)	21 (88%)	7 (50%)
Pulmonary consolidation	9 (4%)	0	0	0	2 (8%)	7 (50%)

*A total of 205- chest CT cases included in this table. Other 13 patients had chest X-ray*.

### Laboratory Indices

Laboratory indices on admission are shown in Table 4. With increasing grades of disease severity based on clinical classification, the proportion of lymphocytes gradually decreased (*p* = 0.001). Elevated D-dimer levels were significantly associated with disease severity (*p* < 0.000), with high D-dimer levels in the severe (0.76 ± 1.22 μg/mL) and critical (1.76 ± 3.34 μg/mL) groups. With increasing grades of disease severity, the level of lactate dehydrogenase gradually increased (*p* = 0.000).

**Table 4:** Initial laboratory results of patients with COVID-19.

	**All patients (*n =* 218)**	**Asymptomatic cases (*n =* 24)**	**Mild cases (*n =* 10)**	**Moderate cases (*n =* 146)**	**Severe cases (*n =* 24)**	**Critical cases (*n =* 14)**	***P*-value**
**Hematologic**							
Leucocytes (×10^9^/L; reference range 3.69–9.16)	5.92 ± 3.23	6.22 ± 2.06	5.19 ± 1.40	5.72 ± 3.18	6.39 ± 3.89	7.24 ± 4.30	0.407
Lymphocytes (×10^9^/L; reference range 0.8–4.0)	1.25 ± 0.61	1.68 ± 0.79^d, e^	1.95 ± 0.67^d, e^	1.26 ± 0.55^d, e^	0.90 ± 0.40^a, b, c^	0.76 ± 0.33^a, b, c^	0.001*
**Coagulation function**							
APTT (s; reference range 23.0–40.0)	33.64 ± 13.51	36.49 ± 13.70^e^	34.86 ± 7.76^e^	31.64 ± 7.45^e^	32.54 ± 4.70^e^	50.37 ± 38.1^a, b, c, d^	0.001*
D-dimer (μg/ml; reference range 0.0–0.7)	0.45 ± 1.06	0.31 ± 0.18^e^	0.212 ± 0.083^e^	0.29 ± 0.22^e^	0.76 ± 1.22^e^	1.76 ± 3.34^a, b, c, d^	0.000*
**Biochemistry**							
Alanine aminotransferase (U/L; reference range 0–40.0)	27.88 ± 21.62	23.54 ± 16.75	18.99 ± 6.92	27.62 ± 20.16	36.90 ± 34.88	24.76 ± 11.76	0.173
Aspartate aminotransferase (U/L; reference range 0–40.0)	27.75 ± 13.55	19.36 ± 7.77^c, d, e^	22.80 ± 6.58^d^	27.37 ± 13.11^a, d^	34.08 ± 17.86^a, b, c^	33.44 ± 11.23^a^	0.004*
Serum creatinine (μmol/L; reference range 53.0–115·0)	72.39 ± 56.64	65.81 ± 21.27	64.83 ± 10.07	67.47 ± 37.33	102.47 ± 137.40	84.66 ± 21.84	0.082
Serum urea (mmol/L; reference range 2.86–7.14)	4.21 ± 3.02	3.89 ± 1.39	3.79 ± 0.48	4.04 ± 3.40	4.95 ± 2.09	5.27 ± 1.75	0.440
Lactate dehydrogenase (U/L; reference range 114.0–240.0)	236.77 ± 216.84	167.47 ± 47.54^e^	172.71 ± 41.18^e^	212.07 ± 76.62^e^	292.90 ± 85.76^e^	510.06 ± 733.24^a, b, c, d^	0.000*
C-reactive protein (mg/L; reference range 0–3.0)	18.57 ± 33.82	1.64 ± 2.34^d, e^	0.95 ± 0.73^d, e^	13.46 ± 23.58^d, e^	38.70 ± 51.53^a, b, c, e^	66.01 ± 54.34^a, b, c, d^	0.000*
**Erythrocyte sedimentation rate** (mm/h; reference range 0–20.0)	41.09 ± 31.72	14.00 ± 19.76^c, d, e^	14.60 ± 21.9^d, e^	43.03 ± 30.24^a^	50.56 ± 31.69^a, b^	61.00 ± 37.98^a, b^	0.000*
**PaO2/FiO2** (mm Hg; reference range 400–500)^†^	NA	NA	NA	NA	NA	176 ± 49	··

*Data are n (%), n/N (%), mean (SD), and median (IQR)*.

* *ANOVA was used for group comparisons with LSD for post-hoc tests*.

*^a^vs. Asymptomatic cases (p < 0.05), ^b^vs. Mild cases (p < 0.05), ^c^vs. Moderate cases (p < 0.05), ^d^vs. Severe cases (p < 0.05), ^e^vs. Critical cases (p < 0.05)*.

† *We analyzed the data when patients were monitored in ICU*.

### Treatment

The chief method of patient management was through symptomatic treatment. Regardless of severity, the vast majority of patients received antiviral treatment. Several patients had bacterial infections and were also given antibiotics. In detail, among the 218 patients, 192 (88%) patients received lopinavir/ritonavir, 192 (88%) patients received interferon-alpha inhalation, 126 (58%) patients received arbidol, 115 (53%) patients received antibiotics, 47 (22%) patients received corticosteroids, 33 (15%) patients received gamma globulin, four (2%) patients received convalescent plasma, and three (1%) patients received umbilical cord mesenchymal stem cell treatment. For respiratory support, 156 (72%) patients were treated with oxygen treatment (including a nasal catheter, mask oxygenation, and/or nasal high-flow oxygen therapy), 16 (7%) with mechanical ventilation, and 14 (6%) with prone position ventilation. Five (2%) patients required renal replacement therapy. Most distinctive is that the majority of cases (196/218, 90%) received traditional Chinese medicine, which is a different treatment approach from that used in other countries. Among these Chinese medicines, the Qingfei Paidu decoction (114/218, 52%) and Lianhuaqingwen capsules (66/218, 30%) were the most frequently used. Huoxiangzhengqi liquid was used only in patients with gastrointestinal discomfort, while the Xuebijing injection was mainly used for severe and critical patients (Table 2).

### Prognosis

There was one death in our cohort of 218 hospitalized COVID-19 patients. This patient had diabetes, hypertension, and severe obesity. As of March 14, most individuals (217/218 [99.5%]) had recovered and were discharged from the hospital. Among the patients who survived, the median hospital stay was 12.2 days (IQR 8–16 days). There were 25 (11%) patients who developed serious conditions during hospitalization, including pulmonary aggravation requiring oxygen ventilation or transfer to an ICU, and 13 patients did not receive steroids during the early stage of the disease but were treated with corticosteroids at a later stage. Nine (<1%) patients had rapid disease progression.

Among the whole cohort, 11% of patients (25/218) were admitted to the ICU and 7% (16/218) received mechanical ventilation. Of the 6% of patients (14/218) diagnosed with ARDS, all belonged to the critical group of cases. Among the 16 patients who received mechanical ventilation, one (6%) died, and the remaining 15 (94%) were discharged before March 14, 2020. Overall, 25 patients in our cohort met the criteria for a poor outcome (death or ICU admission with or without mechanical ventilation). The majority of these poor outcomes occurred within 10 days of hospitalization.

Table 5 shows summaries of the age, sex, clinical classification, and initial laboratory results of patients classified as having a poor prognosis. Univariate analysis of these data showed that advanced age, disease severity (based on clinical classification), an increased activated partial thromboplastin time (APTT), a higher erythrocyte sedimentation rate, and elevated levels of lactate dehydrogenase and C-reaction protein were significantly associated with poor outcome. Lymphopenia was also significantly associated with poor outcome.

**Table 5:** Analysis of poor outcome and clinical features.

	**Univariate analysis, mean (IQR)**		
**Variable**	**No poor outcome (*n =* 193)**	**Poor outcome^†^ (*n =* 25)**	***P*-value**
Age, y	40.9 (30.0–50.0)	58.4 (49.0–67.5)	0.000
Men, %	107 (55%)	15 (60%)	0.831
Clinical classification			0.000
Critical cases	0	14 (56%)	··
Severe cases	13 (7%)	11 (44%)	··
Moderate cases	146 (76%)	0	··
Mild cases	10 (5%)	0	··
Asymptomatic cases	24 (12%)	0	··
Leucocytes, × 10^9^/L	5.87 ± 3.19	6.34 ± 3.50	0.493
Lymphocytes, × 10^9^/L	1.31 ± 0.61	0.80 ± 0.34	0.000
APTT, s	23.89 ± 16.71	39.17 ± 33.99	0.045
ALT, U/L	27.81 ± 22.57	28.40 ± 13.37	0.899
AST, U/L	27.12 ± 13.78	32.22 ± 11.02	0.078
Scr, μmol/L	71.78 ± 59.78	77.00 ± 21.26	0.679
LDH, U/L	211.08 ± 77.18	433.43 ± 574.54	0.000
CRP, mg/L	12.89 ± 24.69	58.84 ± 56.48	0.000
ESR, mm/h	38.51 ± 30.58	62.43 ± 34.03	0.001
	**Univariate analysis**^**‡**^ **Relative risk (95% CI) of poor outcome^§^**		***P*****-value**
Age≥60 y	3.6 (1.6–8.0)		0.001
Diabetes	5.9 (2.7–13.0)		0.000
Other comorbid disease	8.9 (3.0–26.0)		0.000
	**Multivariable analysis**^**‡**^ **Relative risk (95% CI) of poor outcome^§^**		***P*****-value**
Age≥60 y	1.9 (0.8–4.2)		0.134
Diabetes	3.0 (1.3–6.8)		0.007
Other comorbid disease	5.9 (1.9–17.8)		0.002

*Data are median (IQR), n (%), or mean (SD)*.

*APTT, Activated partial thromboplastin time; ALT, Alanine aminotransferase; AST, Aspartate aminotransferase; Scr, Serum creatinine; LDH, Lactate dehydrogenase; CRP, C-reaction protein; ESR, Erythrocyte sedimentation rate*.

† *Defined as death or intensive care unit admission with or without mechanical ventilation*.

‡ *Results are from Cox proportional hazards model*.

§ *Reference group is younger than 60 years, with no diabetes, and no other comorbid disease (chronic pulmonary disease, cardiovascular disease, chronic renal diseases, cerebrovascular disease, liver disease, cancer, malnutrition, or autoimmune disease)*.

Following univariate analysis (Table 5), the Cox proportional hazards model showed that the risk of a poor outcome was increased for those aged 60 years or older [relative risk (RR), 3.6; 95% CI, 1.6–8.0; *p* = 0.001]. The presence of any comorbid disease (other than diabetes) was found to increase the risk of a poor outcome (RR, 8.9; 95% CI, 3.0–26.0; *p* = 0.000), as was the presence of diabetes (RR, 5.9; 95% CI, 2.7–13.0; *p* = 0.000).

Multivariable Cox proportional hazards analysis was performed with the *a priori* hypothesis that age and comorbid diseases were independently associated with a poor outcome (Table 5). In the model including diabetes, other comorbid diseases, and an age ≥60 years, no significant association was found between advanced age and poor outcome (RR, 1.9; 95% CI, 0.8–4.2; *p* = 0.134). However, diabetes alone or with other diseases (RR, 3.0; 95% CI, 1.3–6.8; *p* = 0.007) and any comorbid diseases other than diabetes (cardiovascular disease, chronic pulmonary disease, and other chronic diseases; RR, 5.9; 95% CI, 1.9–17.8; *p* = 0.002) were independently associated with a poor outcome.

**Figure 3:**
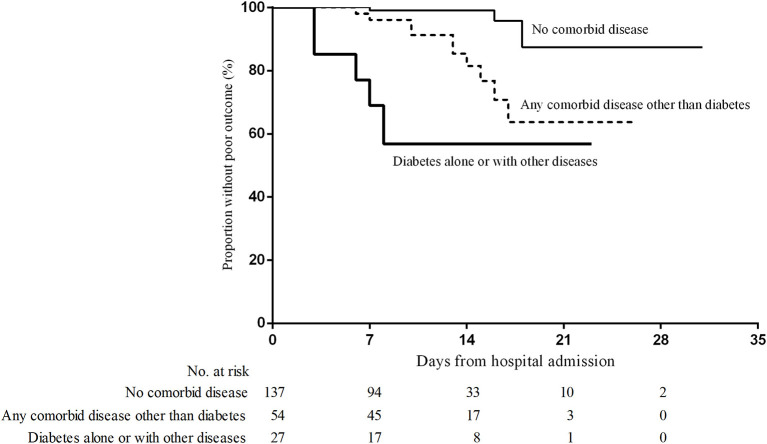
Time from admission to poor outcome based on the presence of a comorbid disease. Poor outcome was defined as death or intensive care unit admission with or without mechanical ventilation.

Despite age ≥60 years, diabetes, and other chronic diseases all being positively associated with a poor outcome, a comparison of the parameter estimates as well as the standard errors in the single and multivariable models indicated that collinearity was not apparent. The standard error for the age parameter was only marginally larger in the multivariable models than in the univariate regression model of age alone. Figure 3 shows the Kaplan–Meier survival curves for these three groups defined by the presence and absence of diabetes and other chronic comorbidities.

### Follow-Up

To date, 20 patients (20/218, 9%) have retested positive for SARS-CoV-2 RNA in nasopharyngeal swabs after having recovered and being discharged. Among these, 18 were classified into the moderate disease group and two were classified into the mild group upon their first admission. These patients showed relatively mild symptoms or were asymptomatic during follow-up. Thus, far, no critical or severe cases have retested positive after being discharged.

## Discussion

Here, the characteristics of a cohort of 218 COVID-19 patients were summarized based on clinical classification of disease severity. This study reflects China's initial experience as the first country to respond to the virus. These findings have important clinical, infection control, and public health implications.

Most patients were clinically classified as moderate cases and had a good prognosis. The median age of the patients increased with the clinical classification of disease severity. Continued vigilance is, therefore, warranted for this high-risk group. The prognosis for the elderly and patients with diabetes and other chronic comorbidities was poor. We attempted to analyze the role of each comorbid disease in COVID-19; however, the number of patients was too small to perform statistical analyses when subgrouping each comorbid disease separately. As for diabetes, previous studies have shown that diabetes can affect the prognosis of patients with viral pneumonia and it should, therefore, be analyzed separately from other comorbid diseases (15). In the univariate analysis performed here and in a previous study by Chen et al. (17), diabetes was found to be associated with poor COVID-19 outcome. For this reason, we analyzed data from the diabetes patients separately from those with other comorbid diseases.

The hallmark laboratory findings of our study indicated that elevated levels of lactate dehydrogenase, C-reaction protein, and D-dimer, as well as an increased erythrocyte sedimentation rate, were positively correlated with clinical classification. Thus, these factors may be involved in disease progression and should receive further attention.

Asymptomatic cases comprised 11% of our cohort, suggesting that there may be a large number of asymptomatic patients in the general population who have not been tested and are transmitting the virus (18). In agreement with a report by Guan et al. who studied a cohort of 1,099 COVID-19 patients in China (6), the most common symptoms reported here were cough, fever, sputum production, and fatigue. Cough was the first symptom reported by many patients (74%). Only 3% of patients had nasal congestion and rhinorrhea, which may assist in differentiating this disease from the common cold.

Most patients had positive CT images. CT imaging has been observed to show multiple ground-glass opacities and even infiltration in both lungs as COVID-19 progresses (19, 20). In severe cases, pulmonary consolidation may be found (19). Chest CT is very important for COVID-19 diagnosis and patient management. Therefore, if medical conditions permit, it is recommended that patients undergo follow-up CT (20).

Currently, no standard treatment has been recommended for coronavirus infection besides careful supportive care (11, 21–23). Given the retrospective nature of our study, it was difficult to determine whether there was any therapeutic benefit conferred by the treatment regimens used for COVID-19, particularly the antibiotic and corticosteroid treatments (24). Treatment with lopinavir/ritonavir was previously reported to show potential in the treatment of SARS, and it can be supposed that this treatment may be beneficial in the treatment of COVID-19 (25).

Recent reports suggest that patients recover from COVID-19 when they receive combined traditional Chinese and Western medicine (23). In our cohort, 53% of patients received antibacterial agents, 88% received antiviral therapy, and 22% received methylprednisolone. Furthermore, 90% received Chinese medicine treatment. The favorable outcome observed for most cases in this cohort may support a COVID-19 treatment approach comprising a combination of traditional Chinese medicine and modern therapies (26). Notably, the most common Chinese medicines, the Lianhuaqingwen capsule and Qingfei Paidu decoction, have proven to be effective in viral pneumonia (27, 28), whereas the Xuebijing injection has been used for severe pneumonia for many years (29).

In agreement with Guan et al. (6), only 7% of the patients in our cohort required mechanical ventilation. Furthermore, we observed a low crude mortality rate (0.5%). This may be related to early nucleic acid detection in close contacts, as well as the relatively low incidence and adequate medical resources found in Hunan province (2). Cases with an exposure history tended to have a milder clinical classification, which may be owing to the vigilance of patients and healthcare workers in seeking early diagnosis and treatment.

Age, lymphocytes, lactate dehydrogenase, C-reaction protein, and erythrocyte sedimentation rate were all associated with the clinical classification. In our multivariable Cox proportional hazards model, diabetes and other chronic comorbid conditions were independently associated with poor prognosis, although an age of 60 years and older was not. Larger sample studies are needed to further elucidate which patients are at most risk of death or requiring admission to an ICU (8).

Currently, the RT-PCR is the standard test for the diagnosis of COVID-19 (11, 30). Notably, the infection appears to be transmitted during the incubation period of the index patient, in whom the illness is brief and non-specific (31). Asymptomatic cases in this study comprised 11% of the patients, all of whom were potential sources of SARS-CoV-2 infection (32, 33). To increase the positive rate of nucleic acid testing, we recommend that sputum and nasopharyngeal swabs be retained as much as possible (11). We further recommend that RT-PCR be repeated twice or more for suspected cases and close contacts as early as possible. This can facilitate early diagnosis, early isolation, and early treatment, and help to reduce the spread of disease (34).

The main strength of our study lies in the application of a new method for clinical classification. Zhang et al. studied the clinical and laboratory characteristics of 140 community-infected COVID-19 patients (35). They compared the data between only severe and non-severe groups, which were defined according to clinical severity. Here, the clinical classification of COVID-19 was performed by referencing the *Diagnosis and treatment protocol for COVID-19 (trial version 7)* (11), which is the latest version of the clinical practice guidelines and has stricter criteria. In this way, the classification and category distribution of groups were described comprehensively and systematically. Using this approach, we found that the moderate cases were the most common. In contrast, the proportions of severe and critical cases were relatively small. In the context of the high prevalence of SARS-CoV-2, the current clinical classification is particularly significant for the guidance of patient management and treatment. Further, our pilot results showed that most of the patients who retested positive for SARS-CoV-2 were from the moderate and mild groups. As such, our classification approach may have implications for clinical monitoring, treatment, and prognosis.

Our study had several limitations. One was the relatively low number of patients and critical cases included. A larger sample size with a greater proportion of critical cases is necessary for future investigations. Moreover, our study was not a randomized controlled trial but rather a retrospective study. Multiple drugs were used, making it difficult to evaluate the effectiveness of a single treatment. Hence, randomized, controlled, multicenter clinical trials are needed to confirm the present findings. As a retrospective observation, the main focus of this study was the nucleic acids present in swabs from the respiratory system. Testing stool nucleic acid is a valuable complementary tool to better understand COVID-19 progression and transmission. Future projects investigating the clinical longitudinal changes in COVID-19 should take the stool nucleic acid test into consideration.

In conclusion, despite the widespread implications of COVID-19, most patients have a favorable clinical prognosis. The COVID-19 epidemic has placed enormous strain on the health and economic status of nations. The excellent spirit of international collaboration among clinicians, researchers, and government agencies needs to continue in an effort to better control and treat COVID-19 (36–38).

## Data Availability Statement

The raw data supporting the conclusions of this article will be made available by the authors, without undue reservation.

## Ethics Statement

The studies involving human participants were reviewed and approved by Ethics Committee of Shaoyang Central Hospital; Ethics Committee of Loudi Central Hospital; Ethics Committee of Xiangtan Central Hospital. Written informed consent to participate in this study was provided by the participants' legal guardian/next of kin.

## Author Contributions

XY and YZ contributed to the study concept and design of this study. XY, XH, DP, YF, ZF, DL, YX, SZ, FC, and WL contributed to the acquisition, analysis, interpretation of data, and the drafting of the paper. YZ contributed to the review and the revision of the manuscript. All authors give final approval to this manuscript for publication.

## Conflict of Interest

The authors declare that the research was conducted in the absence of any commercial or financial relationships that could be construed as a potential conflict of interest.
