# Supplementary material for: Clinical Characteristics and Prognosis of 218 Patients With COVID-19: A Retrospective Study Based on Clinical Classification
Source: Front Med (Lausanne). 2020 Aug 11;7:485. doi: 10.3389/fmed.2020.00485 (PMC7431472; doi:10.3389/fmed.2020.00485)
Supplement: Supplementary file 1 [file Presentation_1.pdf]

Supplementary Material

Detailed information regarding the Clinical Classification and Treatment of the COVID-19 patients included in this study

Clinical Classification

In this retrospective study, the whole disease course was examined for each patient. Cases were classified into four groups based on the clinical conditions relative to the most severe stage, according to the clinical classification of COVID-19 referenced from the *Diagnosis and treatment protocol for COVID-19* (trial version 7), which was released by the National Health Commission of the People's Republic of China on March 3, 2020 (http://www.nhc.gov.cn/xcs/zhengcwj/202003/46c9294a7dfe4cef80dc7f5912eb1989.shtml).

These four classification groups were:

1. Mild cases

The clinical symptoms were mild, with no sign of pneumonia on imaging.

2. Moderate cases

The presence of fever and respiratory symptoms with radiological evidence of pneumonia.

3. Severe cases

Adult cases fulfilled any of the following three criteria: (1) respiratory distress (≥ 30 breaths/min); (2) oxygen saturation ≤ 93% at rest; (3) partial pressure of arterial oxygen (PaO_2)/fraction of inspired oxygen (FiO_2) ≤ 300 mmHg (1 mmHg = 0.133 kPa). In high-altitude areas (> 1,000 meters above sea level), the PaO_2/FiO_2 was corrected using the following formula: PaO_2/FiO_2 × [atmospheric pressure (mmHg)/760]. Cases with chest imaging that showed obvious lesion progression of > 50% within 24–48 hours were also managed as severe cases.

Pediatric cases fulfilled any of the following five criteria: (1) tachypnea (respiratory rate ≥ 60 breaths/min for infants aged < 2 months, ≥ 50 for infants aged 2–12 months, ≥ 40 for children aged 1–5 years, and ≥ 30 for children > 5 years old) independent of fever and crying; (2) oxygen saturation ≤ 92% on finger pulse oximeter taken at rest; (3) labored breathing (moaning, nasal fluttering, along with infrasternal, supraclavicular, and intercostal retraction), cyanosis, and intermittent apnea; (4) lethargy and convulsion; (5) difficulty feeding and signs of dehydration.

4. Critical cases

Pediatric and adult cases fulfilled any of the following three criteria: (1) respiratory failure and requiring mechanical ventilation; (2) shock; (3) other organ failure requiring ICU care.

Treatment

The treatment venue was determined based on the severity of the disease according to the *Diagnosis and treatment protocol for COVID-19* released by the National Health Commission of the People's Republic of China. Suspected and confirmed cases were isolated and treated at designated hospitals with effective isolation, protection, and prevention conditions. Suspected cases were treated in isolation in a single room. Confirmed cases were treated in the same room. In the absence of pathogen-specific interventions, patient management largely depended on supportive treatment.

1. General treatment

Letting patients rest in bed and strengthening support therapy; ensuring sufficient caloric intake; monitoring their water intake and electrolyte balance to maintain internal environment stability; closely monitoring vital signs and oxygen saturation.

Timely provision of effective oxygen therapy, including a nasal catheter, mask oxygenation, and nasal high-flow oxygen therapy.

Antiviral therapy: lopinavir/ritonavir (400 mg twice daily and 100 mg twice daily, respectively), interferon-alpha inhalation (5 million U twice daily), and arbidol (200 mg twice daily).

Some patients were treated with moxifloxacin and other antibiotics to fight against bacterial infection.

For the patients with rapidly progressive deterioration based on imaging results and excessive activation of the body’s inflammatory response, glucocorticoids were used for a short period (3–5 days).

2. Treatment for serious cases

The management of these patients mainly relied on symptomatic therapy, with vigilance for and treatment of complications and secondary infections. Critical cases were admitted to the

ICU and given comprehensive treatment and care from an early stage.

3. Traditional Chinese medicine treatment

(1) Qingfei Paidu decoction

Scope of application: suitable for mild, moderate, and severe patients, and can be used for critical patients depending on their clinical situation.

Usage: decoction in water, one dose per day, twice in the morning and evening (forty minutes after a meal), taken with warm water, three doses a course.

(2) Lianhuaqingwen capsules

Scope of application: fatigue and fever.

Usage: 1.4 g, thrice daily.

(3) Huoxiangzhengqi liquid

Scope of application: gastrointestinal symptoms.

Usage: 10 mL, twice daily.

(4) Xuebijing injection

Scope of application: serious cases.

Usage: start with a small dose and gradually adjust the dose according to the instructions. Recommended usage: 250 mL injection containing 200 mL of 0.9% (w/v) sodium chloride injection and 50 mL of Xuebijing concentrate.
